# A Facile and Green Approach for the Preparation of Silver Nanoparticles on Graphene Oxide with Favorable Antibacterial Activity

**DOI:** 10.3390/nano14171455

**Published:** 2024-09-07

**Authors:** Talia Tene, Stefano Bellucci, Joseth Pachacama, María F. Cuenca-Lozano, Gabriela Tubon-Usca, Marco Guevara, Matteo La Pietra, Yolenny Cruz Salazar, Andrea Scarcello, Melvin Arias Polanco, Lala Rasim Gahramanli, Cristian Vacacela Gomez, Lorenzo S. Caputi

**Affiliations:** 1 Department of Chemistry, Universidad Técnica Particular de Loja, Loja 110160, Ecuador; 2 INFN-Laboratori Nazionali di Frascati, Via E. Fermi 54, 00044 Frascati, Italygahraman.lala@gmail.com (L.R.G.);; 3 Surface Nanoscience Group, Department of Physics, University of Calabria, 87036 Rende, Italylorenzo.caputi@fis.unical.it (L.S.C.); 4 Departamento de Producción, Facultad de Ciencias Exactas y Naturales, Universidad Técnica Particular de Loja, Loja 110160, Ecuador; 5 Grupo de Investigación en Materiales Avanzados (GIMA), Facultad de Ciencias, Escuela Superior Politécnica de Chimborazo (ESPOCH), Riobamba 060155, Ecuador; 6 Faculty of Mechanical Engineering, Escuela Superior Politécnica de Chimborazo (ESPOCH), Riobamba 060155, Ecuador; 7 Department of Information Engineering, Polytechnic University of Marche, Via Brecce Bianche 12, 60131 Ancona, Italy; 8 UNICARIBE Research Center, University of Calabria, 87036 Rende, Italy; 9 Laboratorio de Nanotecnología, Area de Ciencias Básicas y Ambientales, Instituto Tecnológico de Santo Domingo, Santo Domingo 10602, Dominican Republic; 10 Nanoresearch Laboratory, Excellent Center, Baku State University, Baku AZ 1148, Azerbaijan

**Keywords:** graphene oxide, silver nanoparticles, calendula officinalis, seed extract, *E. coli*, *S. aureus*, antibacterial activity

## Abstract

Herein, we introduce a simple precipitation method for preparing graphene oxide–silver nanoparticle (GO/AgNP) composites, utilizing *Calendula officinalis* (*C. officinalis*) seed extract as both a reducing and stabilizing agent. Our research combines the sustainable preparation of graphene oxide (GO) with the green synthesis of silver nanoparticles (AgNPs), aiming to explore the potential of the obtained composite as a novel antibacterial material. To establish a benchmark, the synthesis was also performed using sodium citrate, a conventional reducing agent. The resultant GO/AgNP composites were characterized through several analytical techniques, including scanning electron microscopy (SEM), transmission electron microscopy (TEM), atomic force microscopy (AFM), energy dispersive X-ray spectroscopy (EDS), Raman spectroscopy, X-ray diffraction (XRD), infrared (IR) spectroscopy, and ultraviolet–visible (UV-vis) spectroscopy, confirming the successful functionalization of GO with AgNPs. The antibacterial effectiveness of the composites was systematically assessed against *Escherichia coli* (*E. coli*) and *Staphylococcus aureus* (*S. aureus*), with nanoparticle concentrations spanning from 0 to 250 µg/mL, utilizing mostly disk diffusion and colony-forming unit (CFU) count assays. The AgNPs were characterized by a size range of 15–50 nm. Notably, the GO/AgNP composite prepared using *C. officinalis* seed extract demonstrated superior antibacterial activity at all tested concentrations, outperforming both pure GO and the GO/AgNP composite prepared with sodium citrate. The most pronounced antibacterial effect was observed at a concentration of 32.0 µg/mL. Therefore, this innovative synthesis approach may offer a valuable contribution to the development of new therapeutic agents to combat bacterial infections, suggesting further exploration into antibacterial coatings or potential drug development.

## 1. Introduction

Despite large advancements over recent decades, pathogen-induced infections continue to pose substantial health challenges globally, often aggravated by the transformation and spread of these infections [1]. While antibiotics have long been the basis of combating bacterial infections, owing to their effectiveness even at low doses, their overuse has led to an alarming rise in antibiotic-resistant strains, colloquially termed “super bacteria” [2]. This phenomenon and the environmental concerns associated with antibiotic usage emphasize the urgent need for alternative antimicrobial strategies such as composites for use in wound dressings, medical devices, water purification, textiles, food packaging, and surface coatings [3].

Nanotechnology offers alternative strategies, particularly by developing nanomaterials or nanoparticle-based composites [4]. These advanced materials are gaining recognition as interesting alternatives in the fight against bacterial infections [5]. Their efficacy stems from unique characteristics such as high surface area-to-volume ratios, diminutive grain sizes, and robust chemical activity, all coupled with exceptional thermal stability [6]. Furthermore, the ability to fine-tune the antibacterial properties of nanomaterials by manipulating their structural and morphological features positions them in the face of innovative antimicrobial approaches [7].

Several nanomaterials have been identified as promising candidates for biomedical applications, with graphene and its oxidized variant [8,9], graphene oxide (GO), standing out due to their remarkable efficiency in drug nano-carrier applications [10,11]. Graphene, a two-dimensional sheet of carbon atoms arranged in a hexagonal lattice [12], offers a unique structural foundation. On the other hand, GO diverges slightly, comprising few-layered graphene sheets functionalized with various oxygen-containing functional groups such as hydroxyl, epoxy, carboxyl, and carbonyl [13]. These materials have garnered significant attention over recent years, not only for their high thermal stability and biocompatibility, but also for their quasi-planar structure and superior electrical properties [14].

Silver nitrate (AgNO_3_) has shown its efficacy in clinical applications, primarily attributed to its antimicrobial and antioxidant properties [15]. Beyond these, silver nanoparticles (AgNPs) have garnered acclaim for a broader spectrum of biological activities, encompassing anti-fungal, anti-inflammatory, anti-viral, and anti-angiogenesis properties [16,17,18]. Such versatility extends their utility beyond therapeutic applications. Additionally, AgNPs are increasingly recognized for their roles as biological markers and biosensors, particularly in diagnostic fields [19].

To further support the antibacterial effectiveness of GO and AgNPs, it is crucial to understand the underlying mechanisms at play. The functional groups present in GO are adept at generating electrons, which can catalyze the formation of reactive oxygen species (ROS) [20]. This ability facilitates antibacterial action either through direct physical interactions, where GO nanosheets may compromise bacterial membranes leading to disintegration, or via chemical pathways leveraging oxidative stress or electron transfer between GO and pathogens. In contrast, the antibacterial mechanism of AgNPs primarily involves penetrating the bacterial membrane and interfering directly with DNA, culminating in bacterial cell death [21].

Challenges such as the propensity of AgNPs to aggregate and undertake rapid oxidation pose limitations to their extensive clinical application [22]. Against this backdrop, GO appeared as an important platform for the attachment of AgNPs because its high surface area serves as a support for the attachment and stabilization of metal nanoparticles [23], and due to the presence of oxygen-containing functional groups, GO exhibits a hydrophilic character that makes it a water-soluble material. Indeed, some works have demonstrated superior antibacterial properties when GO is decorated with AgNPs [24], forming a graphene oxide–silver nanoparticle (GO/AgNP) composite.

A critical task in the synthesis of GO/AgNP composite lies in the intricacies of its stepwise preparation process, aimed at achieving the desired composite. A commonly employed technique for GO production is the Hummers method [25], which notably involves the use of strong acids. This method has its drawbacks, such as the release of toxic gases like NO_2_ and N_2_O_4_, and the persistence of residual ions like Na^+^ and NO^3−^, which are challenging to eliminate post-purification [26]. In parallel, the synthesis of AgNPs typically involves the reduction of AgNO_3_ using sodium citrate (Na_3_C_6_H_5_O_7_) [27,28]. However, this process, particularly when scaled up, presents its own set of complications. Excessive exposure to sodium citrate can lead to electrolyte imbalances, potentially inducing symptoms ranging from nausea and vomiting to dizziness, allergic reactions, and confusion. Nowadays, the conventional approach to fabricating GO/AgNP composite involves integrating dispersed GO into the silver nanoparticle synthesis [29,30]. While this method successfully yields GO functionalized with AgNPs, it inherits, unfortunately, the issues associated with the individual preparation of GO and AgNPs.

Recently, we successfully circumvented the issue of toxic gas evolution in the synthesis of GO by adopting a sustainable protocol [31,32]. This strategy has not only reduced environmental concerns, but also yielded a highly effective oxidized graphene precursor, demonstrating efficacy in the removal of pollutants such as methylene blue and mercury(II) [33]. Furthermore, Baghizadeh et al. [34] proved a green synthesis for AgNPs, utilizing the seed extract of Calendula officinalis as an alternative to sodium citrate. Interestingly, Calendula officinalis has been shown to exhibit potent antioxidant properties, which are crucial in the reduction process of Ag cations to AgNPs.

To the best of our knowledge, the synthesis and comprehensive characterization of GO/AgNP composites through the combination of these two sustainable methods, and their consequent application in antibacterial pursuits, remains unexplored. Our study aims to bridge this gap by introducing a simple strategy. Our investigation employs a straightforward precipitation technique, wherein AgNO_3_ is reduced to AgNPs, leveraging either *Calendula officinalis* (*C. officinalis*) extract or sodium citrate (for comparative analysis). Then, the synthesized nanoparticles are incorporated at different concentrations into the GO dispersion. The prepared GO/AgNP composites were characterized using a variety of morphological and spectroscopic techniques. To evaluate their antibacterial efficacy, we employed the disk diffusion test, the determination of the minimum bactericidal concentration (MBC), and the colony-forming unit (CFU) count. *Escherichia coli* (*E. coli,* Gram-negative) and *Staphylococcus aureus* (*S. aureus*, Gram-positive) were selected as the model bacteria for these analyses.

It can be further stressed that our research introduces an innovative and easy approach to synthesizing GO/AgNP composites using *C. officinalis* seed extract as both a reducing and stabilizing agent. This is the first study to combine the green synthesis of graphene oxide and silver nanoparticles using such a method, offering a sustainable alternative to conventional synthesis techniques that often involve toxic chemicals and processes. Additionally, our study systematically compares the antibacterial efficacy of GO/AgNP composites prepared with *C. officinalis* extract versus those prepared with the traditional reducing agent, sodium citrate. Our results demonstrate the superior antibacterial activity of the composites synthesized using the green method, particularly against both Gram-positive and Gram-negative bacteria, thus contributing to the development of new antibacterial materials with potential therapeutic applications.

## 2. Materials and Methods

### 2.1. Materials

All chemicals were utilized as they were given without additional purification. Graphite powder (<150 μm, 99.99%, Sigma Aldrich, St. Louis, MI, USA), H_2_SO_4_ (sulfuric acid, ACS reagent, 95.0–98.0%, Sigma Aldrich), KMnO_4_ (potassium permanganate, ACS reagent, ≥99.0%, Sigma Aldrich), HCl (Hydrochloric acid, ACS reagent, 37%, Sigma Aldrich). H_2_O_2_ (hydrogen peroxide, 30%, Merk, Darmstadt, Germany), AgNO_3_ (silver nitrate, ACS reagent, ≥99.0%, Sigma Aldrich), Na_3_C_6_H_5_O_7_ (sodium citrate dihydrate, ACS reagent, ≥99.0%, Sigma Aldrich), NaCl (sodium chloride, ACS reagent, ≥99.0%).

### 2.2. Preparation of GO

GO synthesis was conducted following our previous works [31,32]. Initially, 3.0 g of graphite powder was introduced into 70.0 mL of concentrated sulfuric acid under stirring to achieve a homogeneous dispersion. Maintaining a temperature below 20 °C, this dispersion was transferred to an ice bath, where 9.0 g of potassium permanganate was carefully added. Subsequently, the mixture was transferred to a water bath to gradually increase the temperature to 50 °C over 30 min, all while stirring continuously.

Once stabilized, 150 mL of distilled water was added to the solution over 20 min, ensuring the temperature did not exceed 90 °C. Then, 500 mL of distilled water was introduced, accompanied by the addition of 15 mL of H_2_O_2_. After 1 h, the resulting precipitate was divided into centrifuge tubes and subjected to washing with a 1:10 solution of HCl and distilled water through multiple centrifugation cycles at 10,000 rpm for 10 min each.

The washed precipitate was then transferred to a Teflon container and dried in an oven at 45 °C for 48 h to obtain graphite oxide powder. Subsequently, the powder underwent sonication for 30 min in 500 mL of distilled water, followed by centrifugation at 500 rpm for 10 min. The resulting precipitate was then dried at 45 °C for an additional 48 h.

### 2.3. Preparation of the Seed Extract

Calendula officinalis seeds used in this study were obtained from the Department of Biochemistry, Escuela Superior Politécnica de Chimborazo. A quantity of 25 g of these seeds was subjected to thorough washing with ultrapure water to remove impurities. The cleaned seeds were then boiled in 100 mL of ultrapure water for 30 min, ensuring the extraction of bioactive compounds. After boiling, the mixture was filtered using Whatman No. 1 filter paper to separate the solid residue from the liquid phase, resulting in a clear seed extract. This extract was employed both as a reducing agent and a stabilizer in subsequent experiments.

### 2.4. Synthesis of Silver Nanoparticles: Approach 1

Pristine silver nanoparticles (AgNPs) were synthesized based on the Turkevich method [35]. Briefly, 150 mg of AgNO_3_ was dissolved in 50 mL of ultrapure water and subjected to reflux for 40 min. Upon reaching boiling temperature, a certain volume of the Calendula officinalis seed extracts (25 mL) was carefully added dropwise. The reaction temperature was maintained within the range of 110 to 130 °C for 30 min. Then, the resultant dispersion of AgNPs underwent a dialysis process for 24 h in ultrapure water to remove residual salts, and subsequently it was stored in a chilled vessel protected from light. The nanoparticles obtained through this approach are labeled as AgNP-1. The measured pH was 6.11.

### 2.5. Synthesis of Silver Nanoparticles: Approach 2

Similarly, a solution was prepared by dissolving 5.0 g of AgNO_3_ in 500 mL of ultrapure water. The mixture was heated at reflux, and when the temperature reached approximately 110 °C, a solution comprising 5.0 g of sodium citrate in 50 mL of ultrapure water was added dropwise. The reaction was kept at 130 °C for 50 min. All other conditions remained constant. The resulting nanoparticles synthesized via this method are labeled as AgNP-2. The measured pH was 6.03.

### 2.6. Preparation of GO/AgNP Composite

To simplify the procedure, the functionalization of AgNPs onto the GO surface begins with the preparation of a GO solution, achieved by dissolving GO in ultrapure water to yield a concentration of 650 µg/mL. Sequentially, aqueous solutions of AgNPs were prepared across a spectrum of concentrations: 32.0 µg/mL, 62.5 µg/mL, 125.0 µg/mL, and 250.0 µg/mL. These aqueous solutions were prepared with the nanoparticles obtained from approaches 1 and 2 (i.e., AgNP-1 and AgNP-2). The selection of 32.0 µg/mL was made to simplify the preparation process and ensure consistency across our experiments. This value was chosen to avoid potential forced linearity in the dose–response relationship, providing a more realistic assessment of the antibacterial efficacy of the as-made materials.

Now, the composite preparation ensued with the mixing/stirring of 10 mL of the GO solution with an equal volume of each nanoparticle concentration. After stirring at 1000 rpm for 15 min, the mixtures were subjected to sonication for 10 min in an ultrasonic bath operating at 400 Hz. Then, an additional 15 min of stirring at 1000 rpm was performed before centrifugation at 13,000 rpm for 10 min to facilitate the separation of the composites. The entire process was conducted at room temperature.

For characterization purposes, we selected samples prepared in AgNP solution with a concentration of 32 µg/mL, as they exhibited superior antibacterial properties irrespective of the reducing agent used (i.e., Calendula officinalis seed extract or sodium citrate). These samples are denoted as GO/AgNP-1 and GO/AgNP-2 for simplicity. The average pH values were 6.07.

### 2.7. Culturing of Escherichia coli

The culture medium used for *Escherichia coli* (*E. coli*) was Luria Bertani (LB) broth, prepared by dissolving 5.0 g/L sodium chloride (NaCl), 5.0 g/L yeast extract, and 10.0 g/L casein peptone in distilled water. This mixture was then autoclaved for sterilization. After preparing the LB agar plates, the *E. coli* strain was inoculated and incubated at 37 °C for 24 h to allow for bacterial growth. Post incubation, colonies of *E. coli* were collected using a previously sterilized loop and suspended in sterile saline or peptone water. Serial dilutions were performed by transferring 1 mL of the bacterial suspension into 9.0 mL of sterile diluent, repeated until the desired dilution level was achieved. The target was to match the turbidity of the final suspension to the 0.5 McFarland standard, which corresponds to approximately 1.5 × 10^8^ CFU/mL. A similar approach is carried out for *Staphylococcus aureus* (*S. aureus*). Note that *E. coli* and *S. aureus* were carefully selected as representative bacterial strains due to their distinct and well-documented differences in cellular structure, particularly in their cell walls, which play a crucial role in their interaction with antimicrobial agents. *E. coli*, a Gram-negative bacterium, possesses an outer membrane that provides additional protection against many antimicrobial agents, whereas *S. aureus*, a Gram-positive bacterium, lacks this outer membrane but has a thicker peptidoglycan layer. These structural differences result in varying degrees of susceptibility to antimicrobial treatments. By evaluating the antibacterial activity of our composites against both *E. coli* and *S. aureus*, we aimed to capture a comprehensive overview of the efficacy of the as-made material across these two major bacterial classifications. This approach not only highlights the broad-spectrum potential of our material in combating diverse bacterial pathogens, but also allows us to present a balanced assessment of its antibacterial properties.

### 2.8. Disk Diffusion Test

Petri dishes containing Mueller–Hinton agar were prepared. A 100 µL aliquot of the bacterial inoculum, standardized to a concentration equivalent to 10^7^ CFU/mL, was evenly spread across the surface of each Petri dish using a sterile spreader to ensure uniform coverage. Subsequently, 5 mm diameter disks, pre-impregnated with 20 µL of six different solutions (32.0 µg/mL, 62.5 µg/mL, 125.0 µg/mL, and 250.0 µg/mL of the composite solution, distilled water as a negative control, and GO solution as a comparison standard), were carefully placed on the agar surface using sterile forceps. Each disk was applied individually to avoid cross-contamination. The Petri dishes were then incubated at 37 °C. The zones of inhibition around each disk were measured at 24 h and 48 h post-incubation to assess the antibacterial activity of the test substances. The inhibition zone was measured under visible light conditions.

### 2.9. Minimum Bactericidal Concentration (MBC)

In MBC, test tubes containing 10 mL of Luria Bertani (LB) broth were prepared. To each tube, 20 µL of the bacterial inoculum, adjusted to a concentration of approximately 1.5 × 10^8^ CFU/mL (matching the 0.5 McFarland standard), was added along with 20 µL of different concentrations of the composite (32.0 µg/mL, 62.5 µg/mL, 125.0 µg/mL, and 250.0 µg/mL). These tubes were then incubated at 37 °C for 24 h, post-incubation; the tubes were examined for turbidity, which indicates bacterial growth due to the presence of suspended particles. Tubes showing no turbidity are indicative of effective bacterial inhibition at the respective composite concentration. However, to confirm bacterial death and not just inhibition, contents from the non-turbid tubes need to be subcultured.

For subculturing, Tryptone Soy Agar plates were used. A 100 µL aliquot from each non-turbid tube was spread on these plates, which were then incubated at 37 °C for an additional 24 h, post-incubation; the plates were examined for bacterial growth. The presence of bacterial colonies indicates that the bacteria were only inhibited but not killed by the composite concentration in the original tube, suggesting that the agent is bacteriostatic rather than bactericidal for that concentration. Conversely, the absence of growth indicates effective bactericidal activity at the tested concentration.

### 2.10. Colony-Forming Unit (CFU) Analysis

For the CFU count, Petri dishes with Mueller–Hinton Agar were prepared. To each dish, a mixture of 100 µL of the bacterial inoculum, standardized to a concentration approximating 1.5 × 10^8^ CFU/mL (equivalent to the 0.5 McFarland standard), and 100 µL of the composite at various concentrations (32.0 µg/mL, 62.5 µg/mL, 125.0 µg/mL, and 250.0 µg/mL) was added. The plates were then incubated for 24 h at 37 °C. Post-incubation, colonies on the agar plates were counted. The counting was facilitated by dividing the plate into quadrants. The colonies in one quadrant were counted, and this number was multiplied by 4 to estimate the total number of colonies per plate. To calculate the CFU per mL, the following equation was used:

(1) $$CFU=\frac{No.\;of\;colonies}{Volume\;of\;inoculum\;plated\;in\;mL}\times dilution\;factor$$

Here, the volume of inoculum plated is 0.1 mL (100 µL), and the dilution factor should reflect any dilutions made to the original bacterial culture before adding it to the composite solution.

### 2.11. Characterization

The absorption spectra of GO, AgNP-1, AgNP-2, GO/AgNP-1, and GO/AgNP-2, were recorded using a Thermo Scientific Evolution 220 spectrophotometer. Fourier-transform infrared spectra were collected using a Jasco FT-IR 4000 spectrometer. Raman spectra were acquired using a Jasco NRS-500 spectrometer with a 532 nm laser wavelength (0.3 mW, 100× objective). X-ray diffraction (XRD) patterns were made using a Panalytical Pro X-ray diffractometer with Cu K irradiation with an acceleration voltage of 60 kV and a current of 55 mA. Spectra data were smoothed using a 7-point moving average. The structure and surface morphology were taken out on a transmission electron microscope (TEM, JEM 1400 Plus, JEOL) operating at 80 kV, and a scanning electron microscope (SEM, JSM-IT100 InTouchScope, JEOL) equipped with a JEOL-made dispersive X-ray spectrometer (EDS) with the accelerating voltage of 15 kV. Atomic force microscopy (AFM) analysis was conducted at room temperature utilizing a NaioAFM system equipped with a SMENA SFC050L scanning head. The measurements were carried out in dynamic force mode, which allows for precise surface characterization under near-operational conditions.

To determine the particle size distribution and the average particle size, TEM images were analyzed. A total of 110 individual AgNPs were measured from the GO/AgNP-1 and GO/AgNP-2 composites. The measured diameters were then used to construct histograms representing the particle size distribution for each composite. The average particle size was calculated by taking the arithmetic mean diameter of the measured particles and then the standard deviation was determined.

## 3. Results and Discussion

**Figure 1:**
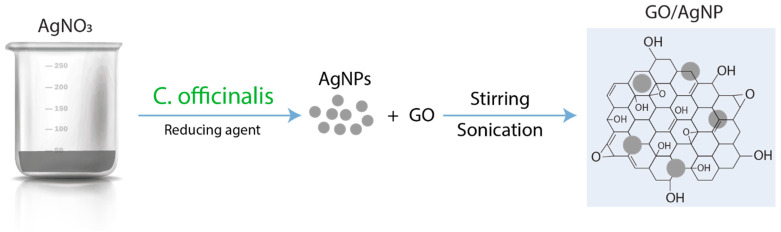
Schematic representation of the synthesis process of GO/AgNP composite.

Figure 1 illustrates the synthesis process adopted in the present investigation (for details see Section 2). AgNPs were synthesized using two distinct approaches: (i) employing seed extract of *C. officinalis* as the reducing agent, we synthesized AgNPs, referred to as AgNPs-1; and (ii) using sodium citrate for the reduction, resulting in the formation of AgNPs denoted as AgNPs-2. Subsequently, GO/AgNP composites were prepared by mixing aqueous solutions of these AgNPs with a GO solution at different nanoparticle concentrations (32.0 µg/mL, 62.5 µg/mL, 125.0 µg/mL, and 250.0 µg/mL). The resultant mixtures were subjected to a combination of stirring and sonication, followed by centrifugation, to induce the uniform distribution of AgNPs on the GO surface.

Interestingly, the GO/AgNP composites demonstrated optimal antibacterial activity at the lowest nanoparticle concentration of 32.0 µg/mL, apart from the reducing agent employed. Hence, these composites at the lowest particle concentration were further characterized and are henceforth referred to as GO/AgNP-1 (using *C. officinalis*) and GO/AgNP-2 (using sodium citrate), respectively.

### 3.1. Morphological and Structural Characterization

**Figure 2:**
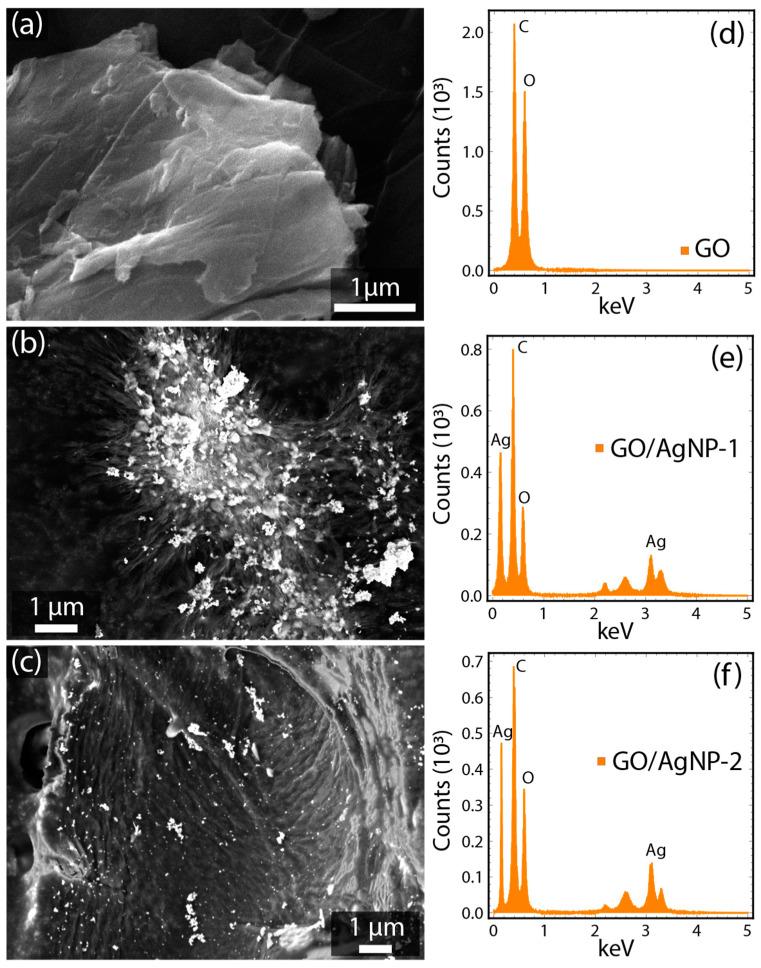
Representative SEM micrographs and EDS spectra of (**a**,**d**) GO, (**b**,**e**) GO/AgNP-1, and (**c**,**f**) GO/AgNP-2.

Figure 2 and Figure S1 present scanning electron microscopy (SEM) micrographs of the synthesized materials, highlighting the morphological distinctions between AgNPs, GO, and related composites. Figure S1a depicts AgNP-1 produced using seed extract of *C. officinalis*, where the nanoparticles demonstrate a homogeneous distribution and reduced agglomeration. Conversely, Figure S1b shows AgNP-2 synthesized with sodium citrate, characterized by a non-uniform distribution and an aggregation propensity [36].

Figure 2a demonstrates the intrinsic morphology of GO, showing a large sheet with characteristic wrinkles and folds [31]. Figure 2b displays the GO/AgNP-1 composite, where the AgNPs are seen to be uniformly dispersed across the GO surface. In Figure 2c, the GO/AgNP-2 composite reveals a more heterogeneous distribution of AgNPs, with distinct clusters and a broader size distribution, indicating a polydisperse assembly on the GO surface. In both cases (i.e., GO/AgNP-1 and GO/AgNP-2 composites), nanoparticle agglomeration is observed. Given this fact, the particle size is estimated from TEM data, while the crystalline size is determined through XRD analysis, as discussed below. It is important to note that particle size and crystalline size are distinct parameters; particle size refers to the overall dimensions of the nanoparticles, including any agglomerated structures, while crystalline size specifically measures the dimensions of the coherent crystalline domains within the particles, which can be smaller or equal than the overall particle size.

The elemental analyses are reported in Table 1 and Table S1. Figure S1c depicts the EDS spectrum for AgNP-1, with prominent silver peaks (Ag: 50.15%) alongside carbon (C: 16:10%) and oxygen (O: 33.75%) signals, suggesting the presence of seed extract as a capping agent. In Figure S1d, the EDS spectrum of AgNP-2 displays a single silver peak (Ag: 100%), indicating that sodium citrate has a low stabilizing influence on the nanoparticles in the current approach.

Otherwise, the EDS spectrum of GO in Figure 2d exhibits the fundamental GO elements, i.e., C and O. Contrastingly, Figure 2e,f reveal the successful functionalization of GO with AgNPs in the GO/AgNP-1 and GO/AgNP-2 composites, as evidenced by the presence of the Ag peak alongside the C and O peaks. It should be noted that the EDS analysis depends on the area exposed to electron bombardment. Therefore, we ensured that the examined region was adequately large to ensure the reliability of these results.

**Figure 3:**
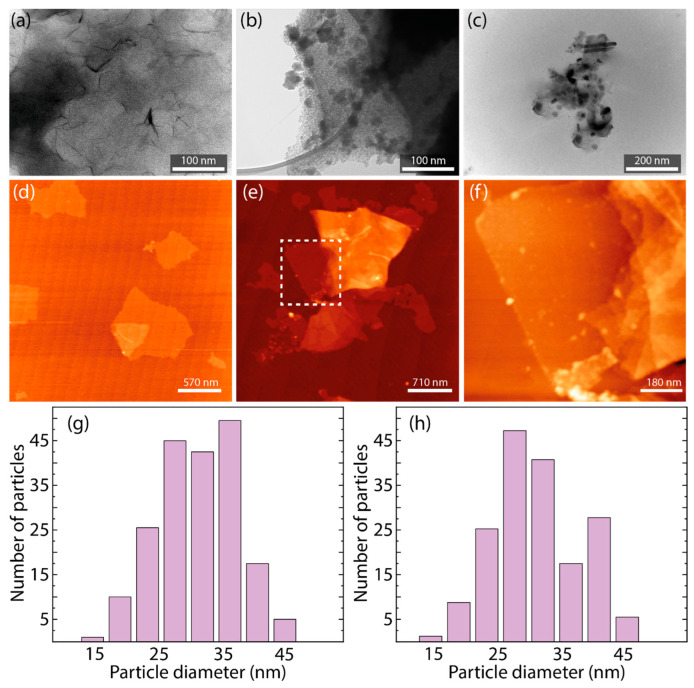
TEM images of (**a**) GO, (**b**) GO/AgNP-1, and (**c**) GO/AgNP-2. AFM images of (**d**) GO, (**e**) GO/AgNP-1, and (**f**) magnified region of GO/AgNP-1 (white-dashed square). Size distribution of silver nanoparticles onto (**g**) GO/AgNP-1 (average size of 31.79 ± 0.70 nm) and (**h**) GO/AgNP-2 (average size of 30.43 ± 1.26 nm).

Figure S2 and Figure 3a–c show the transmission electron microscopy (TEM) images of AgNPs, GO, and their composites. In Figure S2a, AgNP-1 particles are displayed with relative agglomeration, loosely associated, and varying in size, indicating some dispersion with identifiable individual nanoparticles. Figure S2b shows AgNP-2 forming denser clusters and exhibiting greater aggregation, with boundaries between particles less discernible, suggesting a propensity for agglomeration, as observed in SEM results.

Figure 3a presents a TEM image of GO, characterized by a semi-transparent and corrugated nanosheet, covered with characteristic dark wrinkles and folds along the surface and edges. Figure 3b,c feature the GO/AgNP composites. GO/AgNP-1 reveals a remarkably uniform distribution of nanoparticles, suggesting a more synergistic integration with the GO matrix. In contrast, GO/AgNP-2 exhibits a more uneven distribution of nanoparticles with varied densities and a broader spectrum of particle sizes across the GO surface.

Figure S3 and Figure 3d–f present the atomic force microscopy (AFM) images describing the topography of AgNPs, GO, and the GO/AgNP-1 composite (equivalent observations were acquired for the GO/AgNP-2 composite). In Figure S3a, AgNP-1 is depicted with a relatively uniform nanoparticle distribution and a level topography, where the prominent white spots suggest a higher nanoparticle density. Conversely, Figure S3b demonstrates AgNP-2 with a more textured surface, where individual nanoparticles are variably elevated across the substrate, as depicted by the diverse coloration indicating topographical heterogeneity [37].

Figure 3d depicts the GO topography, showcasing the typical smoothness of GO sheets and the varying thickness of the flakes [38]. Figure 3e focuses on the GO/AgNP-1 composite, where nanoparticles can be seen covering the GO surface. The latter is evidenced by the white-dashed square, a specific area enriched with nanoparticles. This area is further examined in Figure 3f, which offers a magnified view into the nanoparticle distribution, with protrusions corresponding to AgNP-1. Notably, the AgNPs were exclusively supported on the GO surface and non-attached nanoparticles were not observed [30,39].

Figure 3g,h describe the particle size distributions for GO/AgNP composites. In Figure 3g for GO/AgNP-1, the histogram reveals a particle size range of 27–35 nm, with an average diameter of 31.79 ± 0.70 nm. Conversely, Figure 3h representing GO/AgNP-2 displays a size distribution with a peak around 28–32 nm and an average diameter of 30.43 ± 1.26 nm. These results suggest a quasi-uniform size among the synthesized nanoparticles.

**Figure 4:**
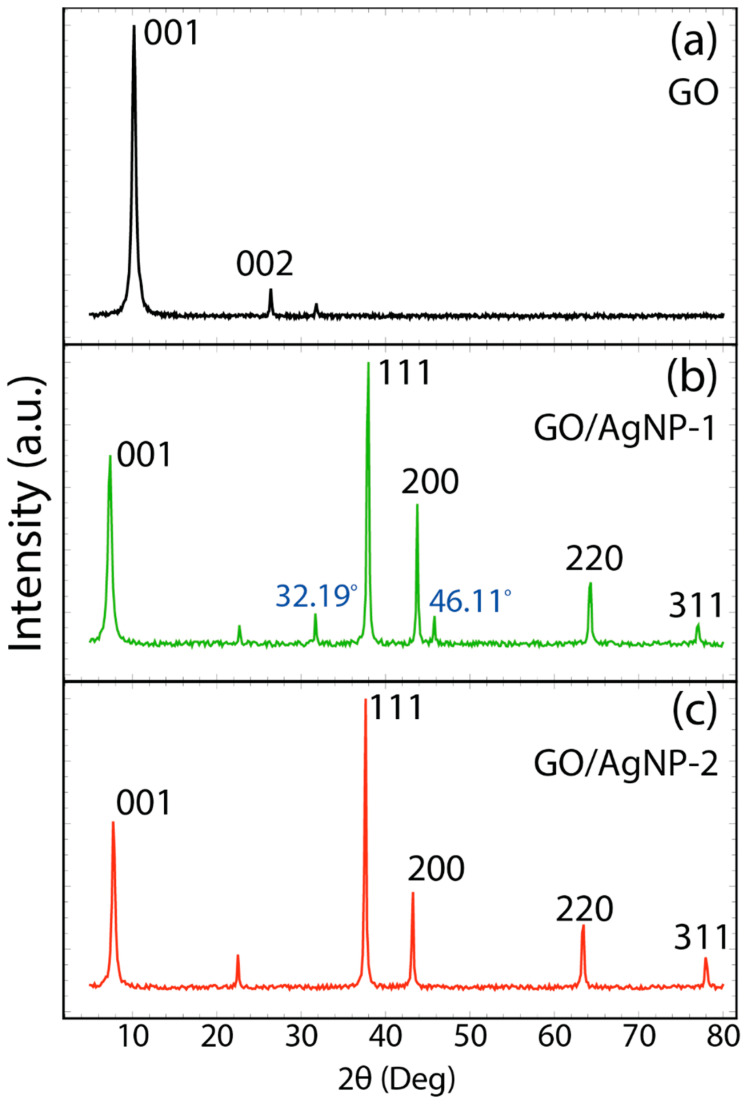
XRD pattern of (**a**) GO (PDF No. 01-0646), (**b**) GO/AgNP-1 (PDF No. 01-0646 for GO and PDF No. 04-0783 for Ag), and (**c**) GO/AgNP-2 (PDF No. 01-0646 for GO and PDF No. 04-0783 for Ag). Spectra data were smoothed using a 7-point moving average.

The structural characteristics of the nanoparticles synthesized were elucidated through XRD analysis. Figure S4 and Figure 4 display the XRD patterns for AgNPs, GO, and their composites. In Figure S4a, the XRD pattern of AgNP-1 reveals prominent peaks at 2θ values of 38.11°, 44.29°, 64.49°, and 77.30°, correlating to the (111), (200), (220), and (311) crystallographic planes of silver, respectively [40]. Additionally, two minor peaks at 32.19° and 46.11°, not typically associated with silver, are observed. These peaks are likely attributed to bioorganic compounds present on the surface of the green-synthesized AgNPs [41,42]. Furthermore, this XRD pattern aligns with the EDS results, confirming silver as the predominant component. AgNP-2, shown in Figure S4b, exhibits a similar XRD pattern with primary peaks at comparable 2θ values. However, it notably lacks the additional unassigned peaks, suggesting a cleaner silver nanoparticle formation.

The average crystalline size of the AgNPs is estimated by using Debye–Scherrer’s equation [43], as follows:

(2) $$D=\frac{K\;\lambda }{\beta \;\cos\theta }$$

where K is a dimensionless shape factor (K=0.9), λ is the wavelength of the X-ray radiation used (λ~0.15 nm), β is the full width at half maximum (FWHM) of the XRD peak, and θ is the Bragg angle at which the X-ray is diffracted.

Through the analysis of the FWHM of the (111) Bragg reflection, measured as ~0.25° for AgNP-1 and ~0.28° for AgNP-2, we calculated average crystallite sizes to be 33.12 ± 0.36 nm and 30.07 ± 0.33 nm, respectively. Under the assumption that in highly crystalline materials the crystallite domains often extend throughout the entire particle, these calculated sizes can be equated to the particle sizes [40]. This point is further validated by the particle size data presented in Figure 3g,h, where the observed sizes closely align with our XRD estimations.

Figure 4a displays the XRD pattern of GO, showcasing a prominent peak at 2θ = 10.11°, indicative of the (001) plane. A secondary, less intense peak at 2θ = 26.41° corresponds to the (002) plane. In GO, oxygen functional groups contribute to an increased interlayer spacing, rising from 0.33 nm in graphite to 0.81 nm in GO [31]. The presence of the peak at 26.41° implies a minimal quantity of non-exfoliated graphite oxide.

Conversely, Figure 4b shows the XRD pattern of the GO/AgNP-1 composite. Here, the functionalization with AgNP-1 is evident and a noticeable shift in the peak position to a lower 2θ value (from 10.11° to 7.31°) is observed, denoting the intercalation of AgNPs within the GO sheets. The resulting interlayer spacing is ~1.27 nm. Similarly, the GO/AgNP-2 composite, shown in Figure 4c, exhibits a peak shift from 10.11° to 7.77°, corresponding to an interlayer spacing of ~1.19 nm [44]. Utilizing Equation (2), the particle sizes for the GO/AgNP-1 and GO/AgNP-2 composites are found to be 32.22 ± 0.35 nm and 30.59 ± 0.34 nm, respectively.

### 3.2. Spectroscopic Characterization

During the reduction process of AgNO_3_ using the seed extract of *C. officinalis*, a notable color transition was observed, shifting from yellowish to brown, as depicted in Figure S5. This color change is an indication of silver nanoparticle formation. Such a transformation is commonly attributed to the excitation of surface plasmon resonances within the AgNPs [45,46].

**Figure 5:**
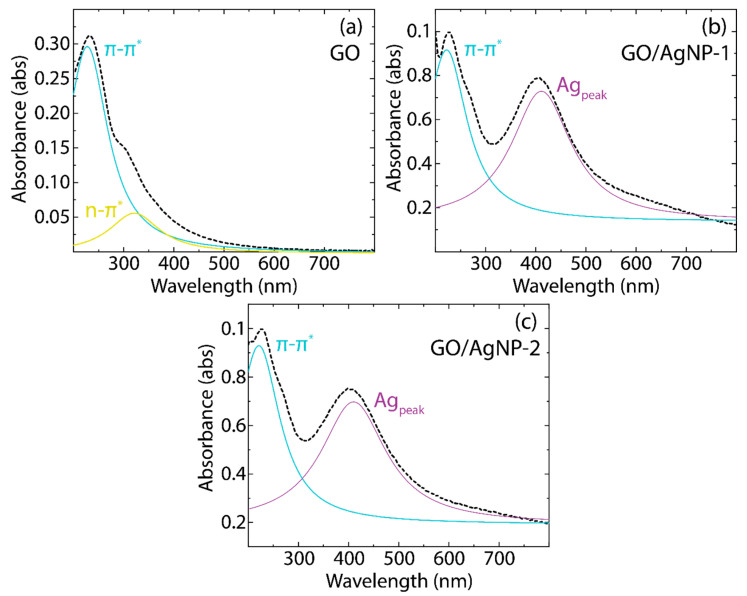
Absorbance spectra (UV-vis) of (**a**) GO, (**b**) GO/AgNP-1, and (**c**) GO/AgNP-2. The spectra data underwent a smoothing employing a 7-point moving average. Intensity normalization was performed relative to the most prominent peak, followed by fitting using two Lorentzian functions.

Figure 5 and Figure S6, along with Table 2 and Table S2, present the absorbance spectra of AgNPs, GO, and their composites, providing details into the position and FWHM of the prominent absorbance peaks. Specifically, Figure 5a displays the GO spectrum, which features two distinct peaks: a primary absorbance peak at ~227 nm (cyan curve) and a shoulder peak at ~321 nm (yellow curve). The first peak is associated with the aromatic $\pi \to \pi^{*}$ transitions of the C–C bonds, while the second peak is attributed to the $n\to \pi^{*}$ transitions typically seen in C=O bonds [47].

Figure S6 displays absorbance peaks at 421 nm for AgNP-1 (Figure S6a) and 434 nm for AgNP-2 (Figure S6b). These peaks are indicative of the main silver absorbance (Ag_peak_) band. On the other hand, the absorbance spectra of the GO/AgNP-1 (Figure 5b) and GO/AgNP-2 (Figure 5c) composites are characterized by two absorbance peaks. The first peak originates from GO (cyan curve), while the second is attributed to the AgNPs (purple curve). Notably, the peak associated with the $n\to \pi^{*}$ transition (yellow curve) is absent, which is attributed to the overlapping with the Ag_peak_ band.

Based on Table 2 and Table S2, the observed blueshift in the $\pi \to \pi^{*}$ transition and Ag_peak_ peak for the GO/AgNP composites suggests that the interaction between GO and AgNPs alters the electronic structure of GO, possibly due to charge transfer phenomena. Additionally, the reduction in FWHM for these composites indicates that the absorbance peaks become sharper upon nanoparticle functionalization. The latter could be due to the ordering of GO by the presence of AgNPs or due to a more homogeneous distribution of nanoparticle sizes in the composites compared to the standalone AgNPs.

**Figure 6:**
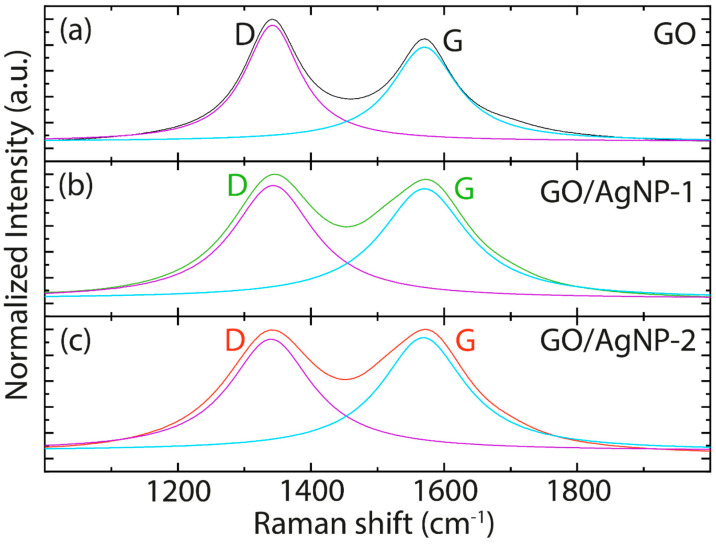
Raman spectra of (**a**) GO, (**b**) GO/AgNP-1, and (**c**) GO/AgNP-2. The spectra data underwent a smoothing employing a 7-point moving average. Intensity normalization was performed relative to the most prominent peak, followed by fitting using two Lorentzian functions (purple and cyan curves).

Figure 6 and Figure S7, and Table 3 and Table S3, show the Raman spectra from 1000 cm^−1^ to 2000 cm^−1^, Raman spectra from 500 cm^−1^ to 3500 cm^−1^, Raman peak position, and Raman FWHM values, respectively. The spectra depicted in Figure S7 highlight the dominant Raman peaks associated with the D, G, and 2D bands characteristic of GO and now observed in GO/AgNP composites. Notably, the intensity of the 2D band in GO is diminished compared to that typically seen in graphene or reduced graphene oxide [48]. This low-intensity 2D band remains largely unaffected upon interaction with AgNPs, suggesting that the presence of the nanoparticles does not alter the structural/layered configuration of GO.

Table 3, and a close view of the Raman spectral region from 1000 cm^−1^ to 2000 cm^−1^ for GO (Figure 6a), reveals that the D band appears at ~1342 cm^−1^ (purple curve) and the G band at ~1571 cm^−1^ (cyan curve). Upon functionalization with AgNP-1 and AgNP-2, these characteristic peaks exhibit negligible shifts, indicating that the interaction with AgNPs does not significantly affect the positions of these Raman bands. However, Table S3 indicates that the Raman FWHM values face a significant alteration upon the addition of AgNPs to GO. For the GO/AgNP-1 composite, there is a notable increase in the FWHM of the D band, rising from ~102 cm^−1^ to ~152 cm^−1^, and for the G band, increasing from ~121 cm^−1^ to ~154 cm^−1^. Similarly, the GO/AgNP-2 composite exhibits an increase in the FWHM of the D band from ~102 cm^−1^ to ~151 cm^−1^, and for the G band, from ~121 cm^−1^ to ~160 cm^−1^. Additionally, there is a variation in the I_D_/I_G_ ratio, decreasing from 1.18 in GO to 1.01 in GO/AgNP-1 and 1.03 in GO/AgNP-2, suggesting changes in the electronic properties within the composites. In particular, the observed changes are primarily ascribed to the surface-enhanced Raman scattering (SERS) effect, which stems from the intense local electromagnetic fields generated by the AgNPs [49]. This enhancement is further augmented by the plasmonic resonance of the silver nanoparticles, which intensifies the Raman signal of the adsorbed molecules on the GO surface, modifying the I_D_/I_G_ ratio.

**Figure 7:**
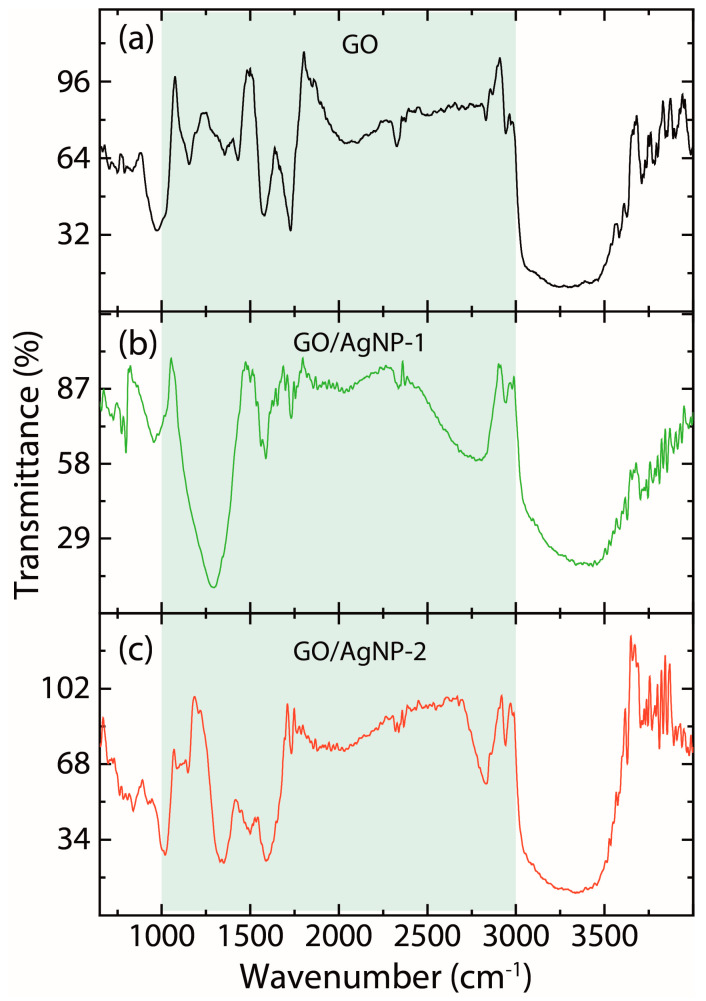
FT-IR spectra of (**a**) GO, (**b**) GO/AgNP-1, and (**c**) GO/AgNP-2. The spectra data underwent a smoothing employing a 7-point moving average. The most important peaks are found in the green region.

The FTIR spectra, along with the corresponding peak positions, are shown in Figure 7 and Table 4, respectively. In the GO and GO/AgNP composites, several functional groups are detected, characterized by distinct vibrational frequencies [16,25,26,39,45,46,47,48]. These include O-H stretching vibrations, observed in the range of 3020 cm^−1^ to 3800 cm^−1^, indicative of hydroxyl groups. The C–H stretching vibrations, characteristic of asymmetric/symmetric hydrocarbon chains, are noted between 2700 cm^−1^ and 2950 cm^−1^. Additionally, C=O stretching vibrations, typically associated with carbonyl groups, appear between 1720 cm^−1^ and 1740 cm^−1^. The spectrum also exhibits peaks for C=C stretching, coming from sp^2^ hybridized C–C bonds in the range from 1570 cm^−1^ and 1600 cm^−1^, and C–O vibrations, noticeable around 1250 cm^−1^. In GO/AgNP-1 (Figure 7b) and GO/AgNP-2 (Figure 7c), extra vibrational bands appear, ranging from 840 cm^−1^ to 3700 cm^−1^, indicating the presence of a capping agent with the nanoparticles [16].

It is noteworthy that following the interaction of pristine GO with AgNPs (GO/AgNP-1 or GO/AgNP-2), the FTIR spectral region most significantly impacted spans from 1000 cm^−1^ to 3000 cm^−1^. In this range, observable alterations in the FTIR spectra include attenuation of some bands, an increase in the intensity of others, or a slight shift in their position. These changes imply the functionalization or intercalation of AgNPs within the GO structure [48], confirming an alteration in the chemical interactions and bonding within the GO/AgNP composites. Details about the detected FTIR bands (assignment and corresponding references) can be seen in Table 4.

### 3.3. Antibacterial Feature

**Figure 8:**
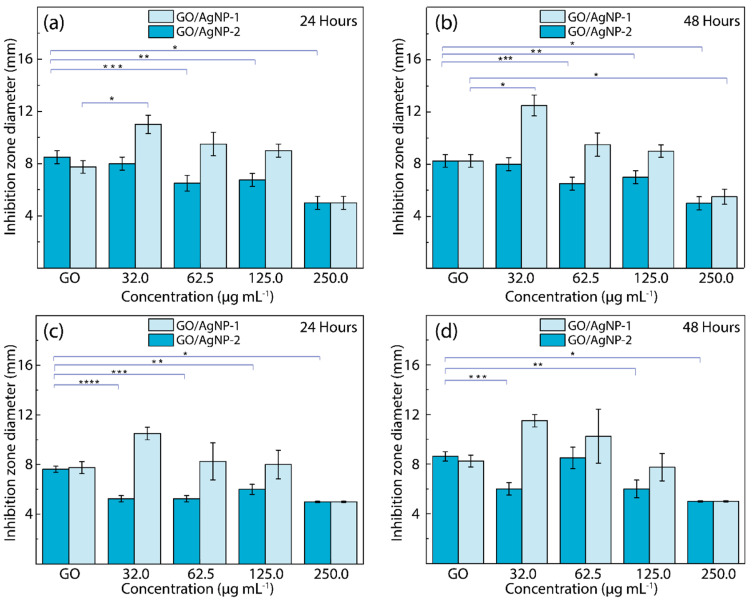
Antibacterial activity of GO, GO/AgNP-1, and GO/AgNP-2 against (**a**,**b**) *E. Coli* and (**c**,**d**) *S. aureus* after exposure to visible light for 24 h and 48 h, respectively, considering different nanoparticle concentrations. The asterisks illustrate statistical significance.

Figure 8 presents the disk diffusion analysis, which highlights the antibacterial potential of GO (control) and GO/AgNP composites. This test quantifies the efficacy of the obtained materials by measuring the diameters of the inhibition zones surrounding 5 mm disks impregnated with GO/AgNP solutions at varying concentrations (see Figure S8, examples of tests carried out). These measurements offer comprehension of the dose-dependent antibacterial performance of the composites. The data are expressed as mean values with standard deviation (SD) derived from four independent experiments. Statistical analysis was performed using a one-way ANOVA, coupled with Dunnett’s post hoc test. Statistical significance is denoted by asterisks, with a *p*-value below 0.05 delineating significant disparities in antibacterial efficacy between the various samples. It is noteworthy that AgNPs exhibit photocatalytic antibacterial properties [49]. As stated, the primary mechanism underlying bacterial killing involves the generation of ROS or free radicals, triggered upon exposure of the nanoparticles to light. This phenomenon has been proven by Hajipour et al. [50] and Menazea et al. [45], who have detailed the interaction mechanism of GO and AgNPs under light irradiation.

In Figure 8a,b, the antibacterial effectiveness of GO/AgNP composites against *E. coli* is evidenced. Regardless of the reducing agent used (*C. officinalis* or sodium citrate), these composites and GO exhibit discernible zones of inhibition, thus demonstrating antibacterial activity at both 24 h and 48 h of exposure. However, our results for GO/AgNP-2 contrast with the findings reported in [45] because we observe a significant decrease (*p* < 0.05) in the inhibition zone diameter for GO/AgNP-2 at varying concentrations compared to GO. Interestingly, GO/AgNP-1 exhibited favorable antibacterial activity at lower nanoparticle concentrations (32.0 µg/mL and 62.5 µg/mL), with a *p*-value of 0.042, signifying an enhanced effect in comparison to GO and GO/AgNP-2. Similarly, Figure 8c,d show the antibacterial effectiveness of the GO/AgNP composites against *S. aureus*. Again, the GO/AgNP-2 composite demonstrates a reduction (*p* < 0.05) in the diameter of the inhibition zones compared to GO across all tested concentrations. In contrast, the GO/AgNP-1 composite does not show statistically significant variations in the inhibition zones across the range of concentrations tested. However, there is an observable increase in the zone of inhibition at a concentration of 32.0 µg/mL.

It is essential to recognize that the synthesis methodology outlined in Ref. [45] diverges from the approach undertaken in this study. Additionally, it is striking that the antibacterial effect exhibited by the composites against both Gram-positive and Gram-negative bacteria diminishes with increasing concentrations of AgNPs at both 24 h and 48 h of exposure. This phenomenon suggests the presence of an optimal antibacterial activity occurring at lower nanoparticle concentrations. Hence, these findings emphasize the critical roles that both the concentration of nanoparticles and the choice of the reducing agent play in modulating the antibacterial efficacy of the GO/AgNP composites. In addition, we hypothesize the following aspects:

- At higher concentrations, AgNPs aggregate (see Figure 2), forming larger clusters that reduce the effective surface area available for interaction with bacterial cells, thereby diminishing antibacterial efficacy.
- The aggregation of AgNPs at higher concentrations leads to a decrease in the available surface area for generating ROS and interacting with bacterial membranes, resulting in lower antibacterial activity.
- Higher concentrations of AgNPs might saturate the bacterial environment, leading to less efficient interactions between nanoparticles and bacterial cells. This could reduce the ability of AgNPs to penetrate bacterial membranes effectively and exert their antibacterial effects.

Interestingly enough, as shown in Table S4, our findings reveal that a concentration of 32 µg/mL of silver nanoparticles alone produces an inhibition zone of 8.0 ± 0.5 mm, regardless of the exposure time (24 or 48 h). This observation emphasizes the superior antibacterial properties of the composite prepared (i.e., GO/AgNP-1) in this study, which outperforms both GO and AgNPs when used separately.

**Figure 9:**
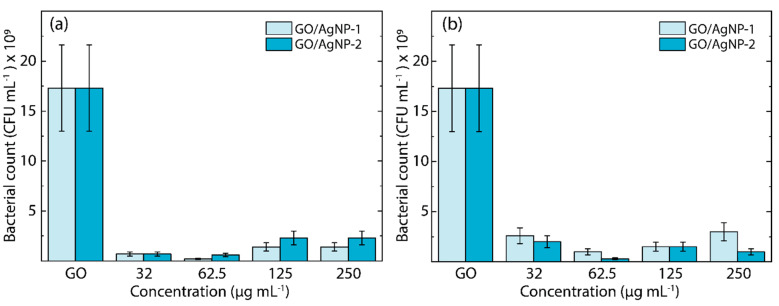
Cell culturability of (**a**) *E. Coli* and (**b**) *S. aureus* exposed to GO/AgNP-1 and GO/AgNP-2 under different nanoparticle concentrations for 24 h. All values are presented as mean ± SD.

On the other hand, in Figure 9, the quantification of colony-forming units (CFUs) offers a direct estimation of viable bacterial cells that are capable of proliferation (see Figure S9). The assay was conducted for both *E. coli* (Figure 9a) and *S. aureus* (Figure 9b) cultures exposed to GO/AgNP-1 and GO/AgNP-2 composites across a range of nanoparticle concentrations (32.0 µg/mL, 62.5 µg/mL, 125.0 µg/mL, and 250.0 µg/mL) for 24 h. Notably, a significant difference in CFU counts was detected between the GO/AgNP composites and GO. The reduction in the number of CFUs is observed across all concentrations.

The CFU assays demonstrate a statistically significant reduction in bacterial viability (*p* < 0.05) when both GO/AgNP-1 and GO/AgNP-2 composites are introduced, as opposed to GO. This statistical trend is consistent across all tested concentrations. The data, presented as mean ± SD and based on a pair of independent experiments, confirm the evidence that nanoparticle concentration is a critical factor influencing the antibacterial effectiveness of the GO/AgNP composites. Particularly, the enhanced antibacterial activity is evident at lower nanoparticle concentrations of 32.0 µg/mL and 62.5 µg/mL for *E. coli*, and at 62.5 µg/mL for *S. aureus*, suggesting an optimal threshold for nanoparticle-mediated inhibition of bacterial growth.

Finally, Tables S5 and S6 summarize the turbidimetric analysis for GO/AgNP-1 and GO/AgNP-2 composites when tested against *E. coli* (Figure S10a) and *S. aureus* (Figure S10b). Turbidity in this context is indicative of bacterial growth, the absence of which suggests effective antibacterial activity [51]. For *E. coli*, at the lowest concentration of 32.0 µg/mL, GO/AgNP-2 exhibits turbidity but no significant growth, while GO/AgNP-1 shows neither turbidity nor growth, indicating a stronger antibacterial effect at this concentration. As the concentration increases, both composites demonstrate effective antibacterial properties against *E. coli*, with no turbidity or growth observed at a concentration of 62.5 µg/mL. In the case of *S. aureus*, similar forms are observed. At 32.0 µg/mL, GO/AgNP-2 shows turbidity without subsequent growth, whereas GO/AgNP-1 exhibits no turbidity, indicating no bacterial growth. These results highlight the bactericidal properties of the GO/AgNP composites, which appear to be more pronounced in GO/AgNP-1 across both bacterial strains, particularly at lower nanoparticle concentrations. These outcomes further stress that the method of nanoparticle synthesis may significantly influence their antibacterial effectiveness.

## 4. Conclusions

In summary, this study reported a green and easy preparation for the synthesis of GO/AgNP composites, employing the seed extract of *C. officinalis* as a dual-purpose reducing and stabilizing agent. For comparative analysis, sodium citrate was also utilized as a reducing agent. The successful functionalization of the GO structure with AgNPs was confirmed via various morphological (SEM, TEM, and AFM) and spectroscopic (EDS, XRD, FTIR, Raman, and UV-vis) characterizations. The GO/AgNP composites synthesized using *C. officinalis* or sodium citrate were called GO/AgNP-1 and GO/AgNP-2, respectively. The synthesized composites were evaluated for their antibacterial effectiveness against model Gram-negative and Gram-positive bacteria, i.e., *E. coli* and *S. aureus*, considering different nanoparticle concentrations (32.0 µg/mL, 62.5 µg/mL, 125.0 µg/mL, and 250.0 µg/mL).

In particular, the antibacterial activity of GO/AgNP composites indicated that both GO/AgNP-1 and GO/AgNP-2 exhibit zones of inhibition, confirming their efficacy. Notably, GO/AgNP-1 shows enhanced antibacterial activity at nanoparticle concentrations of 32.0 µg/mL and 62.5 µg/mL against *E. coli*, and a distinct inhibition increase at 32.0 µg/mL for *S. aureus*. Furthermore, the CFU assay corroborates the reduction in bacterial viability conferred by both composites compared to GO alone, with a significant decrease in CFU counts across all concentrations.

Our study demonstrated that the antibacterial activity of GO/AgNP composites is significantly influenced by nanoparticle concentration and the choice of reducing agent. Indeed, the optimal antibacterial activity is observed at lower nanoparticle concentrations, and the distinctive performance of GO/AgNP-1 indicates that the green synthesis route using *C. officinalis* enhances the antibacterial properties of GO. These insights are critical for further development of GO/AgNP-based antibacterial applications and warrant additional research into the mechanisms driving the observed differences in effectiveness.

## Figures and Tables

**Table 1:** Elemental analysis of GO, GO/AgNPs-1, and GO/AgNPs-2, showing the C, O, and Ag percentage content.

	C (%)	O (%)	Ag (%)
GO	58.12 ± 0.25	41.88 ± 0.36	---
GO/AgNPs-1	53.23 ± 0.29	44.17 ± 0.29	2.60 ± 1.11
GO/AgNPs-2	57.11 ± 0.25	40.82 ± 0.35	2.07 ± 1.03

**Table 2:** The absorbance peak position of the materials under study.

	$\pi -\pi^{*}$	$n-\pi^{*}$	Ag_peak_	R^2^
GO	227.30 ± 0.16	321.22 ± 1.26	---	0.999
GO/AgNPs-1	222.12 ± 9.72	---	411.10 ± 0.74	0.992
GO/AgNPs-2	221.22 ± 0.40	---	410.05 ± 0.47	0.997
AgNPs-1	---	---	421.36 ± 1.30	0.942
AgNPs-2	---	---	433.60 ± 0.51	0.985

**Table 3:** Raman peak position of the materials under study.

	D	G	R^2^
GO	1342.20 ± 0.25	1570.51 ± 0.33	0.982
GO/AgNPs-1	1340.05 ± 0.54	1568.87 ± 0.53	0.973
GO/AgNPs-2	1343.30 ± 0.30	1570.49 ± 0.32	0.992

**Table 4:** FTIR characteristic bands of materials under study.

GO	GO/AgNPs-1	GO/AgNPs-2	Assignment	Ref.
		840.47	N–H deformation of amines	[45]
968.38	949.71		epoxy group	[46]
		1016.93	C–O–C vibrations	[16,26,47]
1150.45		1153.25	C–H in-plane bending	[39]
1250.13	1293.30		C–O vibrations	[25,26]
		1341.85	NO_2_ (N from agNO_3_)	[16]
1353.99			C=O stretching	[48]
1427.75			vibration of carboxyl groups	[45]
1572.46	1591.14	1596.74	C=C stretching	[25]
1727.45	1721.85	1731.19	C=O stretching vibrations	[25,47]
	2788.10			[47]
		2824.51	C–H asymmetric and symmetric stretching vibration	
2940.29	2943.09	2943.09		
3020–3600	3020–3600	3020–3600		
3635.87		3633.07		[25,26]
3705.90	3699.36	3705.90	O–H absorbed water	
3778.72				

## Data Availability

The datasets used and/or analyzed during the current study are available from the corresponding author on reasonable request.

## Supplementary Materials

The following supporting information can be downloaded at: https://www.mdpi.com/article/10.3390/nano14171455/s1, Figure S1. Representative SEM micrographs and EDS spectra of (a,c) AgNP-1 and (b,d) AgNP-2; Figure S2. TEM images of (a) AgNP-1 and (b) AgNP-2; Figure S3. AFM images of (a) AgNP-1 and (b) AgNP-2; Figure S4. XRD pattern of (a) AgNP-1 (PDF No. 04-0783) and (b) AgNP-2 (PDF No. 04-0783). Spectra data were smoothed using a 7-point moving average; Figure S5. Optical images of the synthesis of the silver nanoparticles via the seed extract of Calendula officinalis; Figure S6. Absorbance spectra of (a) AgNP-1 and (b) AgNP-2. Spectra data were smoothed using a 7-point moving average; Figure S7. Raman spectra of (a) AgNP-1 and (b) AgNP-2. Spectra data were smoothed using a 7-point moving average; Figure S8. An example of disk diffusion analysis carried out by measuring the diameters of the inhibition zones; Figure S9. An example of Colony Forming Units (CFUs) analysis; Figure S10. An example of a turbidimetry test subject to the GO/AgNP-1 composite for 24 h at a concentration of 32.0 µg/mL. (a) *E. coli* cultures. (b) *S. aureus* culture; Table S1. Elemental analysis of AgNP-1 and AgNP-2, showing the C, O, and Ag percentage content; Table S2. Absorbance FWHM peak of the materials under study; Table S3. Raman FWHM peak of the materials under study. The ID/IG ratio denotes the intensity ratio of the D peak to the G peak; Table S4. Antibacterial activity of GO, AgNP-1, and GO/AgNP-1 composite against *E. coli* and *S. aureus*, considering different nanoparticle concentrations; Table S5. Turbidimetry analysis of GO/AgNP-1 and GO/AgNP-2 against *E. coli*; Table S6. Turbidimetry analysis of GO/AgNP-1 and GO/AgNP-2 against *S. aureus*.
