# Supplementary material for: A Facile and Green Approach for the Preparation of Silver Nanoparticles on Graphene Oxide with Favorable Antibacterial Activity
Source: Nanomaterials (Basel). 2024 Sep 7;14(17):1455. doi: 10.3390/nano14171455 (PMC11397097; doi:10.3390/nano14171455)
Supplement: Supplementary file 1 [file nanomaterials-14-01455-s001.zip › nanomaterials-3160895-supplementary.pdf]

SUPPORTING INFORMATION:

A Facile and Green Approach for the Preparation of Silver Nanoparticles on Graphene Oxide with Favorable Antibacterial Activity

Talia Tene [1,*], Stefano Bellucci [2,*], Joseth Pachacama [3], María F. Cuenca-Lozano [4], Gabriela Tubon-Usca [5], Marco Guevara [6], Matteo La Pietra [2,7], Yolenny Cruz Salazar [3,8], Andrea Scarcello [3,8], Melvin Arias Polanco [9], Lala Rasim Gahramanli [2,10], Cristian Vacacela Gomez [2,8] and Lorenzo S. Caputi [3,8]

[1] Department of Chemistry, Universidad Técnica Particular de Loja, Loja 110160, Ecuador
[2] INFN-Laboratori Nazionali di Frascati, Via E. Fermi 54, 00044 Frascati, Italy
[3] Surface Nanoscience Group, Department of Physics, University of Calabria, 87036 Rende, Italy;
[4] Departamento de Producción, Facultad de Ciencias Exactas y Naturales, Universidad Técnica Particular de Loja, Loja 110160, Ecuador
[5] Grupo de Investigación en Materiales Avanzados (GIMA), Facultad de Ciencias, Escuela Superior Politécnica de Chimborazo (ESPOCH), Riobamba 060155, Ecuador
[6] Faculty of Mechanical Engineering, Escuela Superior Politécnica de Chimborazo (ESPOCH), Riobamba 060155, Ecuador
[7] Department of Information Engineering, Polytechnic University of Marche, Via Brecce Bianche 12, 60131 Ancona, Italy
[8] UNICARIBE Research Center, University of Calabria, 87036 Rende, Italy
[9] Laboratorio de Nanotecnología, Area de Ciencias Básicas y Ambientales, Instituto Tecnológico de Santo Domingo, Santo Domingo 10602, Dominican Republic
[10] Nanoresearch Laboratory, Excellent Center, Baku State University, Baku AZ 1148, Azerbaijan
* Correspondence: tbtene@utpl.edu.ec (T.T.) bellucci@lnf.infn.it (S.B.)

1. **Supplementary Figures**

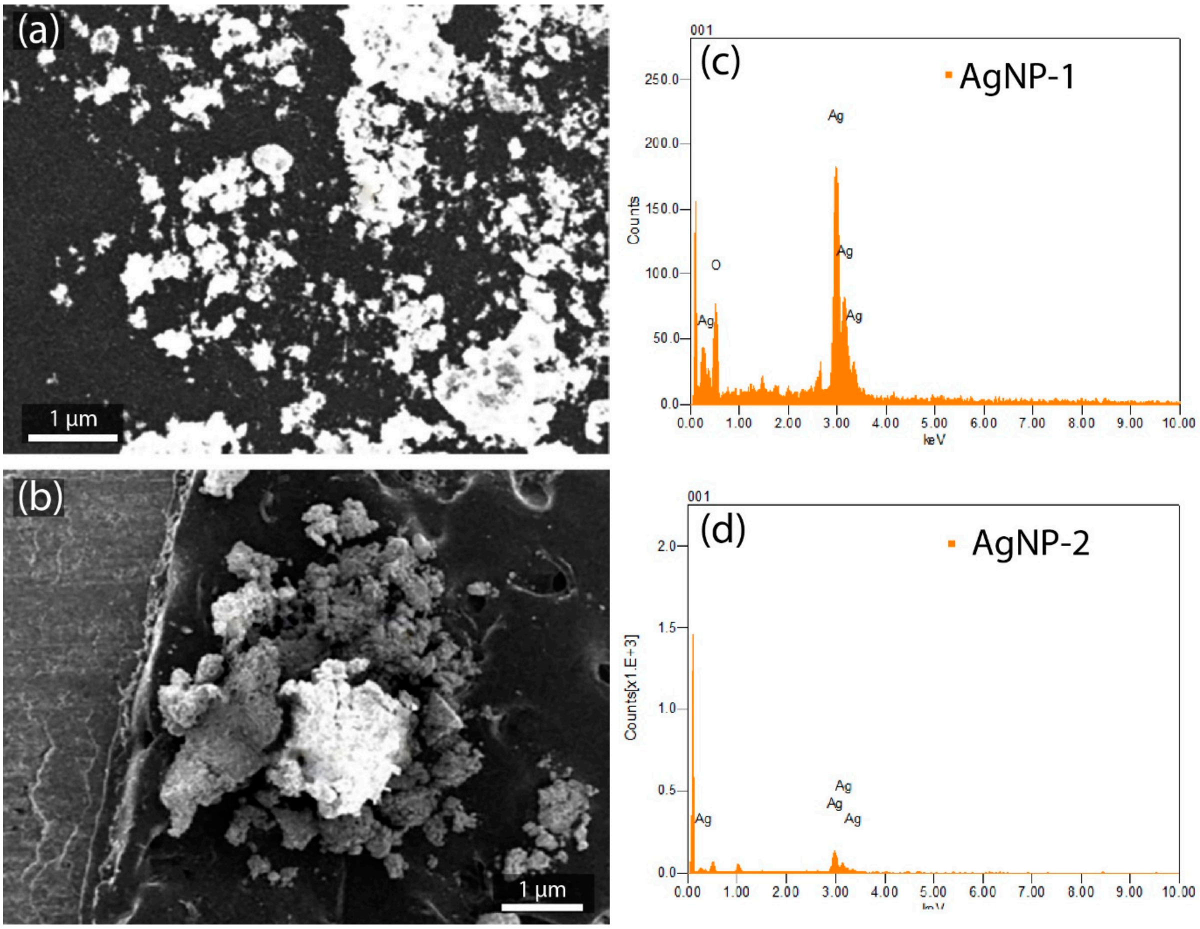

Figure S1. Representative SEM micrographs and EDS spectra of (a, c) AgNP-1 and (b, d) AgNP-2.

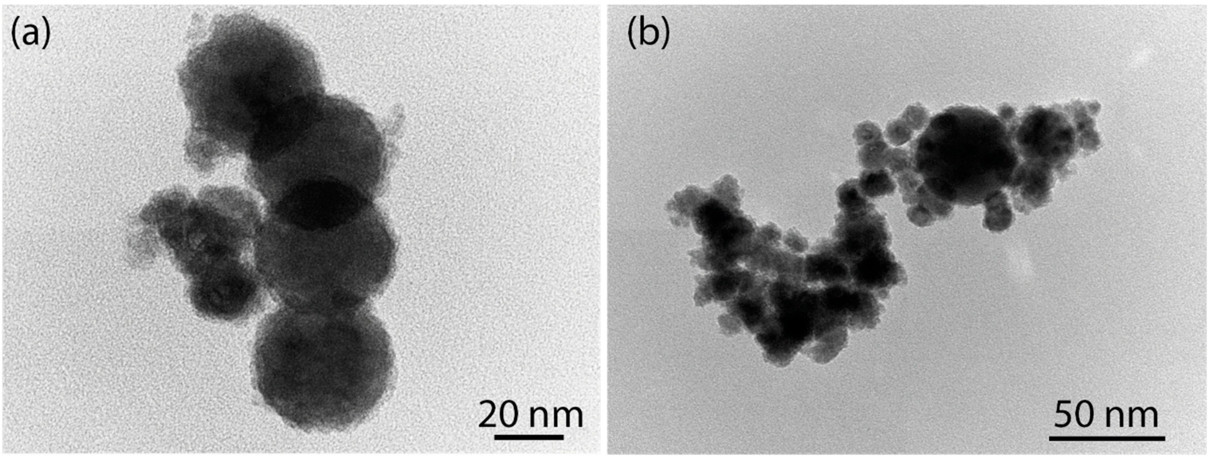

Figure S2. TEM images of (a) AgNP-1 and (b) AgNP-2.

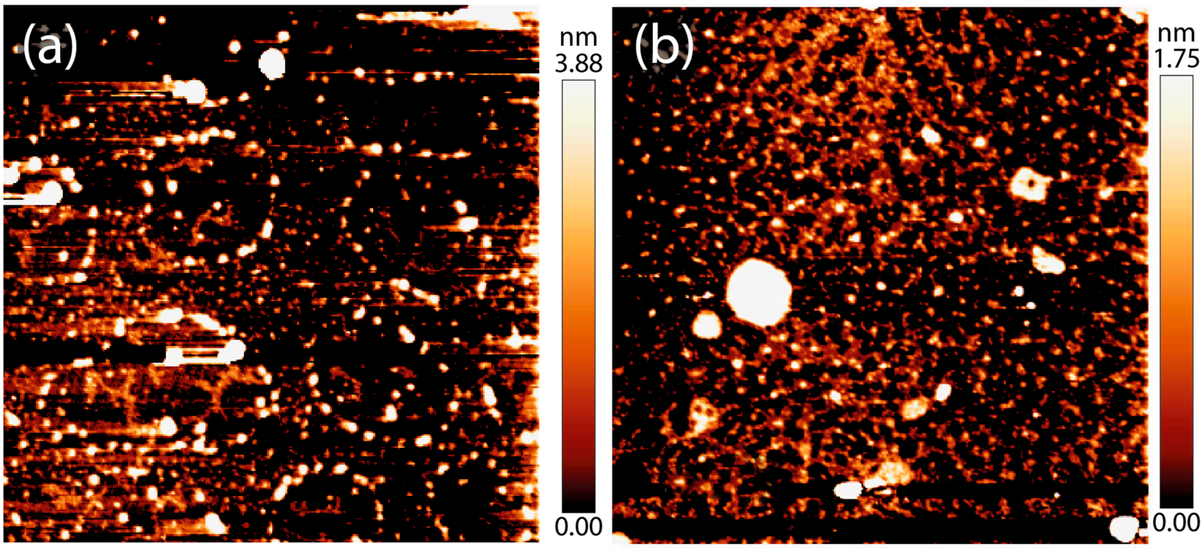

Figure S3. AFM images of (a) AgNP-1 and (b) AgNP-2.

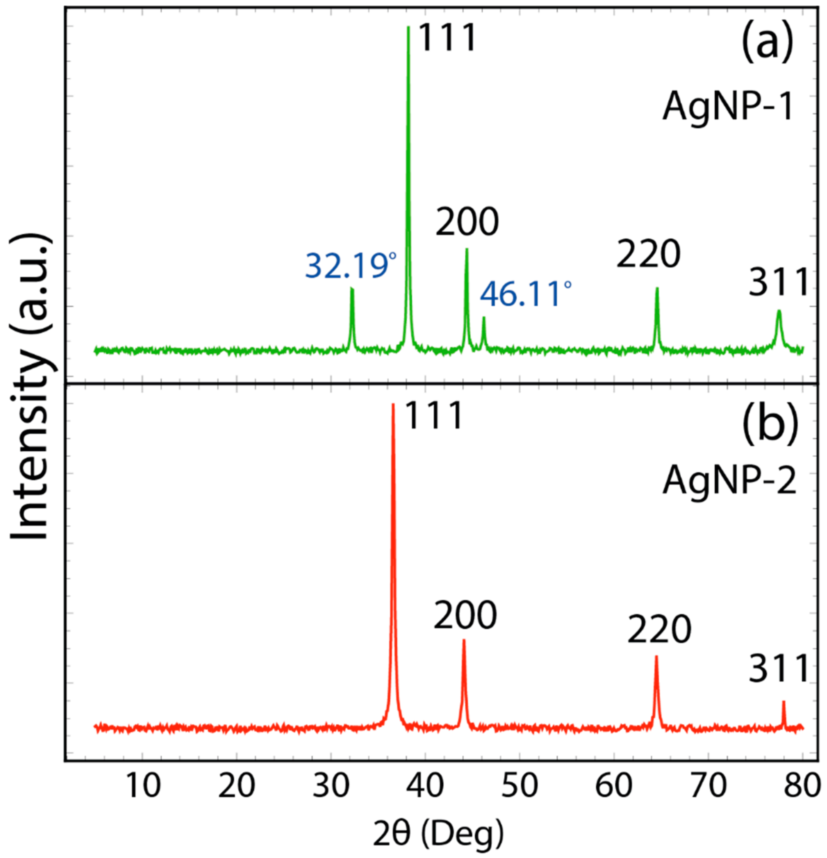

Figure S4. XRD pattern of (a) AgNP-1 (PDF No. 04-0783) and (b) AgNP-2 (PDF No. 04-0783). Spectra data were smoothed using a 7-point moving average.

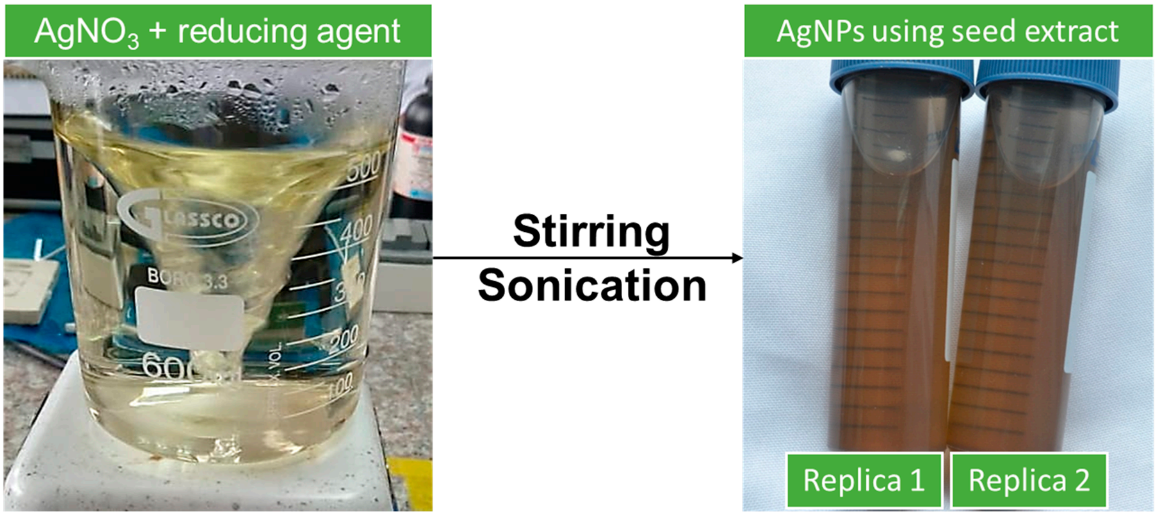

Figure S5. Optical images of the synthesis of the silver nanoparticles via the seed extract of Calendula officinalis.

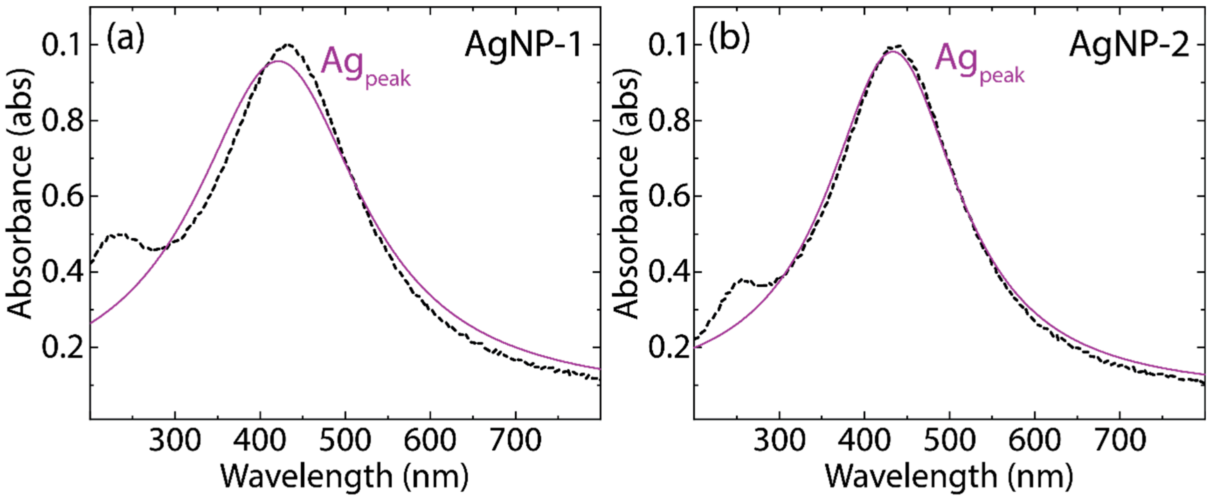

Figure S6. Absorbance spectra of (a) AgNP-1 and (b) AgNP-2. Spectra data were smoothed using a 7-point moving average.

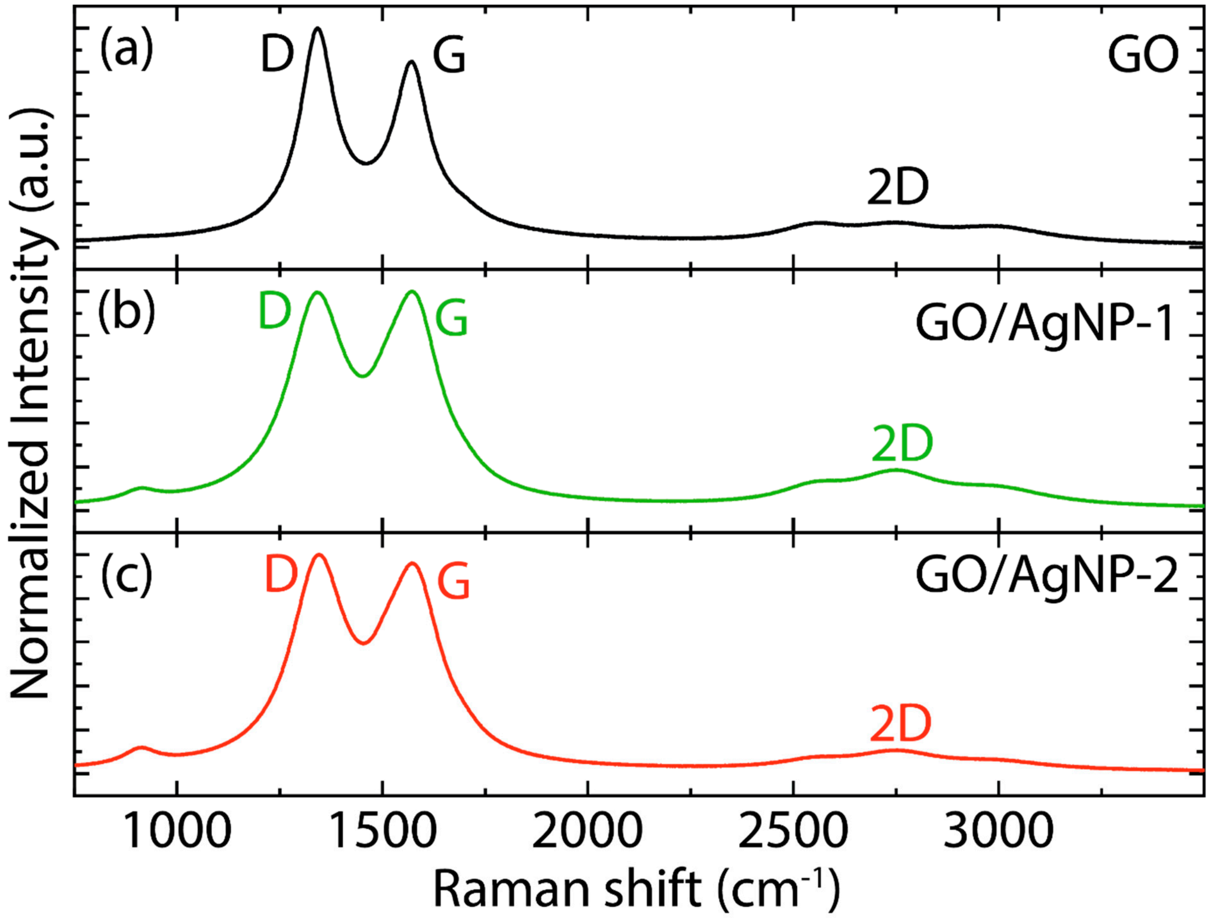

Figure S7. Raman spectra of (a) AgNP-1 and (b) AgNP-2. Spectra data were smoothed using a 7-point moving average.

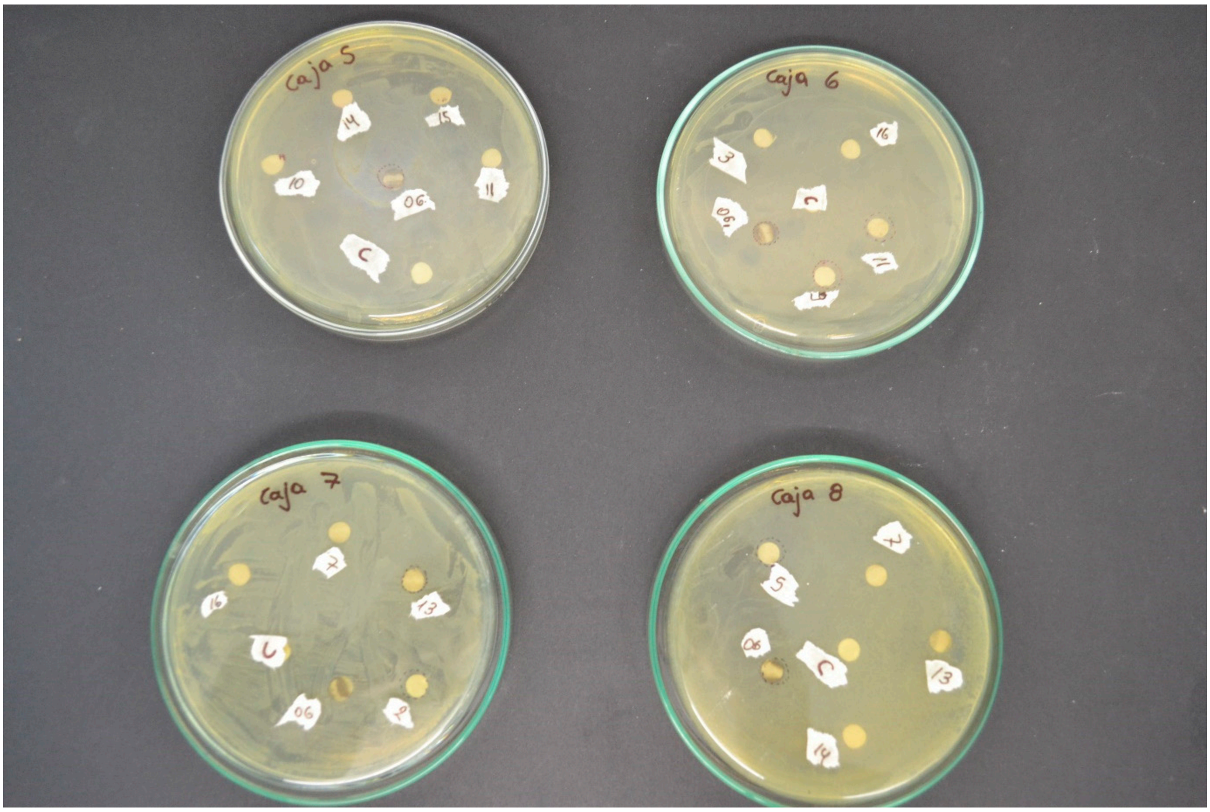

Figure S8. An example of disk diffusion analysis carried out by measuring the diameters of the inhibition zones.

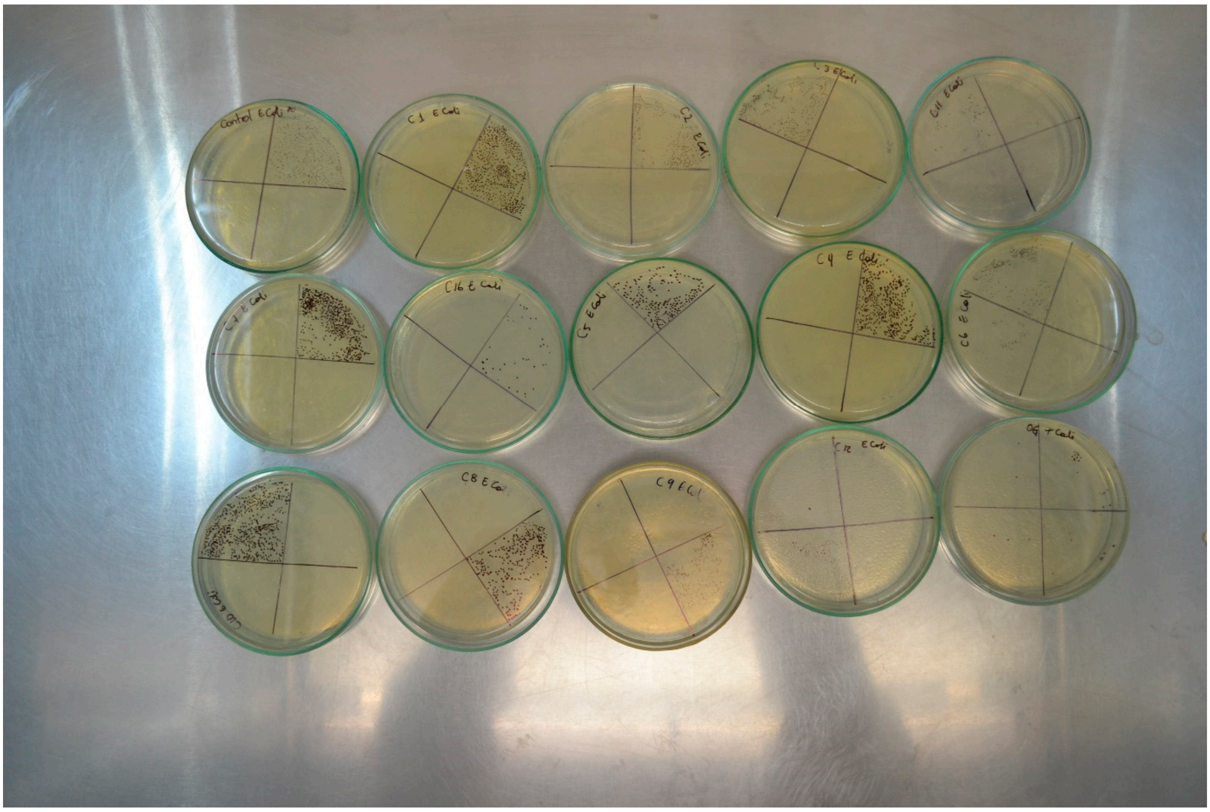

Figure S9. An example of Colony Forming Units (CFUs) analysis.

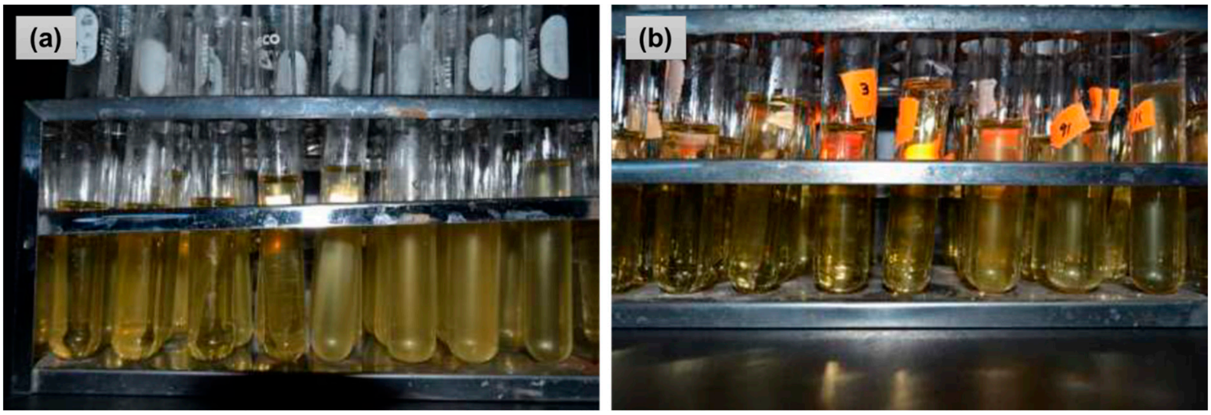

Figure S10. An example of a turbidimetry test subject to the GO/AgNP-1 composite for 24 h at a concentration of 32.0 ug/mL. a) *E. coli* cultures. b) *S. aureus* culture.

2. Supplementary Tables

Table S1. Elemental analysis of AgNP-1 and AgNP-2, showing the C, O, and Ag percentage content.

	**C (%)**	**O (%)**	**Ag (%)**
AgNPs-1	16.10 ± 0.89	33.75 ± 0.47	50.15 ± 0.28
AgNPs-2	---	---	100

Table S2. Absorbance FWHM peak of the materials under study.

	$\pi - \pi^*$	$n - \pi^*$	**Ag_{peak}**	**R^2**
GO	99.99 ± 1.06	121.78 ± 3.17	---	0.999
GO/AgNPs-1	98.77 ± 2.26	---	146.04 ± 2.57	0.992
GO/AgNPs-2	101.86 ± 1.49	---	158.30 ± 1.90	0.997
AgNPs-1	---	---	238.58 ± 7.32	0.942
AgNPs-2	---	---	188.63 ± 2.82	0.985

Table S3. Raman FWHM peak of the materials under study. The I_D/I_G ratio denotes the intensity ratio of the D peak to the G peak.

	**D**	**G**	**I_D/I_G**
GO	102.41 ± 0.78	121.19 ± 1.07	1.18
GO/AgNPs-1	152.21 ± 1.79	153.92 ± 1.79	1.01
GO/AgNPs-2	151.24 ± 0.99	160.12 ± 1.06	1.03

Table S4. Antibacterial activity of GO, AgNP-1, and GO/AgNP-1 composite against *E. Coli* and *S. aureus*, considering different nanoparticle concentrations.

GO/AgNP-1					
		E. Coli		*S. Aureus*	
		24 h	48 h	24 h	48 h
System	Concentration of Nanoparticles (μg/mL)	Inhibition zone (mm)	Inhibition zone (mm)	Inhibition zone (mm)	Inhibition zone (mm)
GO	0.0	7.75 ± 0.48	8.25± 0.48	7.75± 0.48	8.25± 0.48

AgNPs	32.0	8.00 ± 0.50	8.00± 0.50	8.00± 0.50	8.00± 0.50
GO+ AgNPs	32.0	11.00 ± 0.70	12.50± 0.80	10.50± 0.50	11.50± 0.50
GO+ AgNPs	62.5	9.50 ± 0.90	9.50± 0.90	8.25 ± 1.49	10.25± 2.17
GO+ AgNPs	125.0	9.00 ± 0.50	9.00± 0.48	8.00± 1.16	7.75± 1.11
GO+ AgNPs	250.0	5.00 ± 0.50	5.50± 0.58	5.00± 0.05	5.00± 0.05

Table S5. Turbidimetry analysis of GO/AgNP-1 and GO/AgNP-2 against *E. Coli*.

**Concentration (ug/mL)**	**GO/AgNP-2**				**GO/AgNP-1**			
	**Turbidity**		**Growth**		**Turbidity**		**Growth**	
	**YES**	**NOT**	**YES**	**NOT**	**YES**	**NOT**	**YES**	**NOT**
32.0	x		x			x		x
62.5	x		X			x		x
125.5	x		x		x		x	
250.5	x		x		x		x	

Table S6. Turbidimetry analysis of GO/AgNP-1 and GO/AgNP-2 against *S. aureus*.

**Concentration (ug/mL)**	**GO/AgNP-2**				**GO/AgNP-1**			
	**Turbidity**		**Growth**		**Turbidity**		**Growth**	
	**YES**	**NOT**	**YES**	**NOT**	**YES**	**NOT**	**YES**	**NOT**
32.0	x		x			x		x
62.5		x		x		x		x
125.5	x		x		x		x	
250.5	x		x		x		x	
